# Comparative efficacy and safety of traditional Chinese patent medicine for NAFLD in childhood or adolescence: A protocol for a Bayesian network meta analysis

**DOI:** 10.1097/MD.0000000000024277

**Published:** 2021-01-22

**Authors:** Yuli Sun, Zhaofeng Tan, Zhenyuan Jiang, Min Li, Weiqin Wang, Yaoyao Huang, Jianguang Sun

**Affiliations:** a First College of Clinical Medicine, Shandong University of Traditional Chinese Medicine; b Affiliated Hospital of Shandong University of Traditional Chinese Medicine, Jinan, Shandong Province, China.

**Keywords:** NAFLD in childhood or adolescence, network meta-analysis, protocol, TCPM

## Abstract

**Background:**

Nonalcoholic fatty liver disease is a common reason for chronic liver disease in children and adults. The increasing incidence of the disease has become one of the most critical public health problems in the 21st century, closely related to genetic and environmental factors. So far, apart from changing lifestyle and diet, modern medicine still lacks effective treatment measures. Chinese patent medicine has the advantages of apparent curative effect, overall regulation and fewer side effects. However, there is a lack of research on the simultaneous comparison of various Chinese patent medicines. Therefore, we used a reticular meta-analysis to indirectly compare the efficacy and safety of different oral Chinese patent medicines through standard reference.

**Method:**

We will conduct a comprehensive and systematic search of Chinese and English databases from the beginning to December 2020. All randomized controlled trials (RCTs) of oral Chinese patent medicine for NAFLD in children will be searched. The 2 researchers then independently filter the retrieved literature, extract the data according to the data extraction table and assess the risk of bias. We will perform a pair of meta-analyses and a Bayesian network meta-analysis. STATA and Win BUGS software will be used for data analysis.

**Results:**

This study will thoroughly compare and analyze the differences in the efficacy of all kinds of TCPM in NAFLD treatment in childhood or adolescence.

**Conclusion:**

This study will provide reference and evidence support for clinical drug selection optimization.

**Ethics and dissemination:**

This study does not require ethical approval.

**INPLASY registration number:**

2020120068.

## 1 Introduction

Nonalcoholic fatty liver disease (NAFLD) in childhood or adolescence refers to the chronic fatty degeneration of the liver under 18, involving more than 5% of liver cells, drinking, and other apparent pathogenic factors that lead to chronic fatty deposits in the liver are excluded. According to the clinical and pathological characteristics, NAFLD includes nonalcoholic fatty liver (NAFL), nonalcoholic steatohepatitis (NASH) and associated hepatic fibrosis and cirrhosis.^[1]^ NAFLD prevalence in American children is about 3% to 11%, lower than in Asia^[2]^ The morbidity of NAFLD in obese and overweight children has increased significantly, reaching 50% to 80%, which has become a familiar cause of chronic liver disease in children.^[3]^ The increasingly severe epidemic of NAFLD among children is of great concern because NAFLD is considered a more widespread and potentially metabolic manifestation of liver dysfunction and is closely associated with many metabolic risk factors, including insulin resistance, dyslipidemia, cardiovascular disease and obesity.^[4–6]^ Although it is rare to find children die of NAFLD, the risk of hepatic cirrhosis and hepatocellular carcinoma increases significantly in adulthood.^[4,7–9]^ At the same time, it may also increase the incidence and mortality of extrahepatic diseases such as cardio-cerebrovascular diseases in NAFLD.^[10,11]^ Therefore, the prevention and treatment of NAFLD has become a significant public health problem globally and has attracted much attention.

The etiology of NAFLD in childhood or adolescence is very intricate, which is closely related to hereditary and environmental factors. Studies have found that if parents have evidence of hepatic steatosis, their children have a significantly increased risk of fatty liver disease.^[12]^ Some researchers have found that some gene variations encoding proteins regulating lipid metabolism are related to fatty liver. For example, mutations of PNPLA3I148 M and TM6SF2 E167K affect the remodelling of lipid droplet and the secretion of lipid in liver cells, which may increase the risk of liver disease in children under 18;^[13–15]^ The Mttp variant rs2306986 is an independent risk factor for NAFLD onset in children aged 6 to 18 years.^[16]^ It is generally believed that the intestinal liver axis may enhance the interaction between intestinal bacteria-bacterial products and liver receptors, thereby promoting oxidative stress, insulin resistance, liver inflammation and fibrosis. Studies^[17]^ suggest fatty liver. It is related to changes in the diversity of intestinal flora by antibiotic exposure. Therefore, intestinal microecological disorders may also lead to liver steatosis.

Despite the increasing incidence of NAFLD in childhood or adolescence worldwide, modern medicine still lacks specific treatment methods. Currently, the only recognized way is to adjust diet and lifestyle and control body mass. However, its effect is not satisfactory, and it is not easy to be accepted and persisted by children and adolescents.^[18–20]^ At the same time, the results of drug treatment are not satisfactory. A study on vitamin E and metformin for 96 weeks to treat children and adolescence with NAFLD suggesting that the main results of patients with a continuous decline in ALT levels, vitamin E and metformin are not superior to comfort Agent.^[21]^ In another study, metformin, probiotics,ω-3 fatty acids and cysteamine tartaric acid intervened in children with NAFLD and confirmed that its beneficial effects are limited.^[22]^ Since the discovery of fatty liver, traditional Chinese medicine therapy has been used for a long time in China. Chinese patent medicine (TCMP) effectively improves patients clinical symptoms, enhances liver function indicators, prevents recurrence, and other aspects. It has reliable curative effects, overall regulation, high safety and fewer side effects. Therefore, more and more attention has been paid to hepatologists. The proprietary Chinese medicines for the treatment of fatty liver include Xiaoyao Pills, Dangfei Liganning Capsules, Huazhi Rougan Granules, Shell Zhi Capsules, Xuezhikang Capsules, Gynostemma Glycoside Tablets, Yinzhihuang granules, Hugan Ning tablets, Anluo chemical fibre, Liuwei Wuling tablets^[23]^ and so on. However, some clinical observations have analyzed the availability of different proprietary Chinese medicines. However, the efficacy of TCPM has not been compared. Therefore, Chinese patent medicine is more effective for children with NAFLD? Or which proprietary Chinese medicine is suitable for which type of NAFLD? Therefore, a Bayesian network meta-analysis was performed to provide ample and conclusive evidence further.

## 2 Methods and analysis

We will use Bayesian NMA. Then we compliant PRISMA-P guidelines to conduct this study.

### 2.1 Research registration

This mesh meta-analysis study has been registered in Inplasy. The registration number is: INPLASY2020120068 (URL = https://inplasy.com/inplasy-2020-12-0068/).

### 2.2 Inclusion criteria

#### 2.2.1 Types of studies

All RCTs related to clinical observation tests that use TCPM to treat NAFLD in children will be included. The meta-analysis, case reports, reviews, and tasks with insufficient data will be excluded. The language only needs English and Chinese, while in the blind method, there is no limit to the test time.

#### 2.2.2 Participants

The population includes NAFLD in childhood or adolescence diagnosed according to any internationally or nationally authorized diagnostic criteria (such as NAFLD guidelines in children and adolescents; expert consensus on diagnosing and treating NAFLD in children.^[1,24–27]^ There are no restrictions on gender, race, region or other characteristics. Limit gender, race, area, or other features.

#### 2.2.3 Interventions

The experimental group received TCPM alone, or TCPM combined with conventional Western medicine. TCPM included Xiaoyao Pills, Dangfei Liganning Capsules, Huazhi Rougan Granules, Qiao Zhi Capsules, Xuezhikang Capsules, Gynostemma Glycoside Tablets, Yinzhihuang granules, Hugan Ning tablets, Anluo Huaxian Pills, Liuwei Wuling tablets etc; The control group received conventional Western medicine or diet exercise treatment. RCTs using 2 or more TCPM or combined acupuncture, moxibustion, and other traditional Chinese medicine therapies need to be excluded.

#### 2.2.4 Outcomes

The dominating outcome is the rate of remission of clinical symptoms after treatment; alanine aminotransferase (ALT) continues to decrease, defined as 50% or less of the baseline level or 40 U/L or less. The secondary outcome indicators are to improve the histological characteristics of NAFLD and the resolution of NASH; the incidence of liver fibrosis, cirrhosis, and liver cancer; the incidence of diabetes and hypertension.

### 2.3 Search method and retrieval strategies

We will search the CNKI database, Wanfang database, Weipu database, and Chinese biomedical literature database. The search terms are “Proprietary Chinese medicine”, “capsule”, “pellet”, “powder”, “tablet”, “nonalcoholic fatty liver disease”, “nonalcoholic steatohepatitis”, “fatty liver”. At the same time, search Cochrane library, PubMed, Embase, Clinical Trials foreign databases. According to the pre-search, we will adjust the search strategy according to the evolution of the evidence search method and the function of different databases and optimize the search strategy. The retrieval time is up to December 2020. Table 1 shows the initial search strategy for the PubMed database.

**Table 1:** Detailed search strategy for PubMed.

No.	Search item
#1	“Non-alcoholic Fatty Liver Disease” [Mesh Terms]
#2	Steatohepatitis, Nonalcoholic [Title/Abstract] OR Steatohepatitides, Nonalcoholic [Title/Abstract] OR Nonalcoholic Steatohepatitides [Title/Abstract] OR Nonalcoholic Steatohepatitis [Title/Abstract] OR Nonalcoholic Fatty Livers [Title/Abstract] OR Nonalcoholic Fatty Liver [Title/Abstract] OR Livers, Nonalcoholic Fatty [Title/Abstract] OR Liver, Nonalcoholic Fatty [Title/Abstract] OR Fatty Livers, Nonalcoholic [Title/Abstract])) OR Fatty Liver, Nonalcoholic [Title/Abstract] OR Nonalcoholic Fatty Liver Disease [Title/Abstract] OR NAFLD [Title/Abstract] OR Non alcoholic Fatty Liver Disease [Title/Abstract]
#3	#1 OR #2
#4	(Adolescents [Mesh Terms]
#5	Children [Title/Abstract] OR Childhood [Title/Abstract] OR Youths [Title/Abstract] OR Youth [Title/Abstract] OR Teenager[Title/Abstract]OR Teenagers [Title/Abstract] OR Teen[Title/Abstract]OR Teens [Title/Abstract] OR Adolescence [Title/Abstract]
#6	#4 OR #5
#7	complementary Therapies [Mesh]
#8	complementary medicine [Title/Abstract] OR alternative therapies [Title/Abstract] OR medicine, alternative [Title/Abstract] OR Chinese patent medicine [Title/Abstract] OR Chinese proprietary medicine [Title/Abstract] OR Chinese herbal drugs [Title/Abstract] OR herbal [Title/Abstract]
#9	Xiaoyao Pills [Title/Abstract] OR Dangfei Liganning Capsules [Title/Abstract] OR Huazhi Rougan Granules [Title/Abstract] OR Qiao Zhi Capsules [Title/Abstract] OR Xuezhikang Capsules[Title/Abstract] OR Gynostemma Glycoside Tablets [Title/Abstract] OR Yinzhihuang granules [Title/Abstract] OR Hugan Ning tablets [Title/Abstract] OR Liuwei Wuling tablets [Title/Abstract] OR Anluo Huaxian Pills [Title/Abstract]
#10	#7 OR #8 OR #9
#11	Randomized controlled trial [Publication Type] AND Controlled clinical trial [Publication Type]
#12	Randomized [Title/Abstract] OR random allocation [Title/Abstract]
#13	#11 OR #12
#14	#3 AND #6 AND #10 AND #13

### 2.4 Study selection

Note Express was used for literature management. After removal, the literature was selected according to the established inclusion criteria, and 2 researchers were chosen to extract them independently. If there were any objections, the third researcher would assist in the judgment.

### 2.5 Data extraction

We will design a data extraction table to extract data. The content includes title, author, publication time, age, gender, race, sample size, baseline characteristics, interventions, course of treatment, outcome indicators, adverse events, etc.

### 2.6 Evaluation of bias

Two researchers conducted a quality assessment in strict accordance with the risk bias assessment tool recommended by the Cochrane Handbook Version5.3. Topics include: randomization, allocation concealment, blinding of researchers and subjects, blind evaluation of study results, the integrity of outcome data, selective reporting bias, and other biases. The quality of the literature was rated as “high risk,” “low risk,” and “accountability risk.”

### 2.7 Statistical analysis

Stata software is a complete data processing, statistical calculation and drawing software system. We will use Win BUGS in Stata software to carry out Bayesian statistical analysis, and Bayesian inference will be carried out using the Markov chain-Monte Carlo (MCMC) method. The odd ratio (OR) was used as the effect analysis statistic for dichotomous variables. In contrast, the mean difference (MD) was used as the effect analysis statistic for continuous variables, and 95% confidence intervals (CI) were provided for each effect. By using the data results obtained from WinBUGS software called Stata 14.0 software, the ranking products of each intervention were obtained, and the cumulative probability ranking diagram was drawn. The area under the curve (Surface under the Cumulativeranking, SUCRA) was obtained. The SUCRA value is expressed as a percentage. A SUCRA value of 100% indicated that the intervention was absolutely effective. Based on SUCRA values, cluster analysis of outcome indicators was carried out to obtain the 2 cluster indicators relatively best intervention measures.

### 2.8 Assessment of heterogeneity

*I*^2^ judged the heterogeneity, and if (*P* ≥ .10 and *I*^2^ ≤ 50%), the heterogeneity among the studies is slight, and the fixed effect model will be used, followed by reticular Meta-analysis. If (*P* < .1 and *I*^2^ > 50%), we will analyze heterogeneity sources. We will use subgroup analysis or sensitivity analysis for the apparent clinical heterogeneity, exclude the heterogeneity factors or used random effect model combination analysis, and use descriptive analysis for those sources of heterogeneity that can not be found.

### 2.9 Subgroup analysis

If the data are abundant, the heterogeneity will be dealt with by subgroup analysis according to different design schemes, study quality, characteristics of participants, treatment duration, publication age, etc.

### 2.10 Sensitivity analysis

Sensitivity analysis will be implemented through the R2WinBUGS package of R software to verify the clinical and methodological similarities between the included studies and determine the reliability of this study's results.

### 2.11 Publication bias

The effective rate, clinical symptom relief rate, ALT and histological improvement were taken as indicators. The inverted funnel plot was made with each study effect as the abscissa and the standard error of impact as the ordinate. If the inverted funnel chart is not entirely symmetrical, it indicates the possibility of publication bias or a small sample effect.

### 2.12 Hierarchy of evidence quality

The GRADE (Grades of Recommendations Assessment, Development and Evaluation) system was used to assess the quality of evidence, including bias risk, indirect risk, inconsistency risk, imprecise risk and publication bias.^[28]^

## 3 Discussion

NAFLD in childhood or adolescence is relatively insidious, characterized by liver fat accumulation, liver inflammation, and fibrosis. If not timely and effective intervention, it may develop into liver cirrhosis or even liver cancer29. Besides, NAFLD is a mutual cause and effect of obesity and type 2 diabetes, jointly promoting the high incidence of extrahepatic malignancies such as cirrhosis, HCC, cardiovascular disease, chronic kidney disease and colorectal cancer. It will increase the incidence and mortality of complications of extrahepatic organs and increase the risk of death. However, there is still a lack of effective treatment measures in addition to changing lifestyle and diet. In China, TCPM has been used to treat NAFLD for more than 30 years. Proprietary Chinese medicines are effective prescriptions that have undergone long-term clinical verification. They are produced under the strict supervision of relevant departments, including tablets, pills, granules, capsules and other different dosage forms. The preparation process is rigorous and scientific and convenient to take. Chinese people deeply love them. In this study, we will bring in reticular Mate analysis based on the existing RCT to estimate the validity and safety of TCPM to provide evidence support for clinicians. The majority of children and adolescents are the future of the world. We expect that this study will offer clinicians better choices. Reduce liver lipid deposition, improve liver inflammation, and reduce liver fibrosis and liver cancer incidence. It is expected that our research may have some potential limitations. First of all, the potential heterogeneity may stem from the different dosage forms and treatment courses of Chinese patent medicines. Second, there may be some small-sample studies, which may lead to a high risk of bias. Third, including only Chinese and English studies may lead to publication bias. After a comprehensive analysis, the above restrictions will be described in detail in this review's discussion section.

## Author contributions

**Conceptualization:** Yuli Sun, Jianguang Sun.

**Data curation:** Yuli Sun, Zhaofeng Tan, Zhenyuan Jiang, Jianguang Sun.

**Formal analysis:** Yuli Sun.

**Funding acquisition:** Yuli Sun.

**Investigation:** Yuli Sun.

**Methodology:** Yuli Sun, Zhaofeng Tan, Zhenyuan Jiang, Jianguang Sun.

**Project administration:** Zhaofeng Tan, Min Li, Weiqin Wang.

**Resources:** Yuli Sun, Zhenyuan Jiang, Jianguang Sun.

**Search strategy:** Yuli Sun, Jianguang Sun, Zhenyuan Jiang.

**Software:** Yuli Sun, Zhaofeng Tan, Min Li, Yaoyao Huang.

**Statistical analysis:** Yuli Sun, Zhaofeng Tan, Weiqin Wang.

**Supervision:** Yuli Sun, Weiqin Wang, Yaoyao Huang.

**Validation:** Yuli Sun, Weiqin Wang.

**Visualization:** Yuli Sun.

**Writing – original draft:** Yuli Sun.

**Writing – review & editing:** Yuli Sun, Jianguang Sun.
